# Thermally Activated Delayed Fluorescence Coinage Metal Cluster Scintillator

**DOI:** 10.1021/acscentsci.3c00563

**Published:** 2023-06-24

**Authors:** Qiu-Chen Peng, Yu-Bing Si, Zhao-Yang Wang, Shu-Heng Dai, Qiu-Shui Chen, Kai Li, Shuang-Quan Zang

**Affiliations:** † Henan Key Laboratory of Crystalline Molecular Functional Materials, Henan International Joint Laboratory of Tumor Theranostical Cluster Materials, Green Catalysis Center and College of Chemistry, Zhengzhou University, Zhengzhou 450001, China; ‡ MOE Key Laboratory for Analytical Science of Food Safety and Biology, State Key Laboratory of Photocatalysis on Energy and Environment, College of Chemistry, Fuzhou University, Fuzhou 350100, China

## Abstract

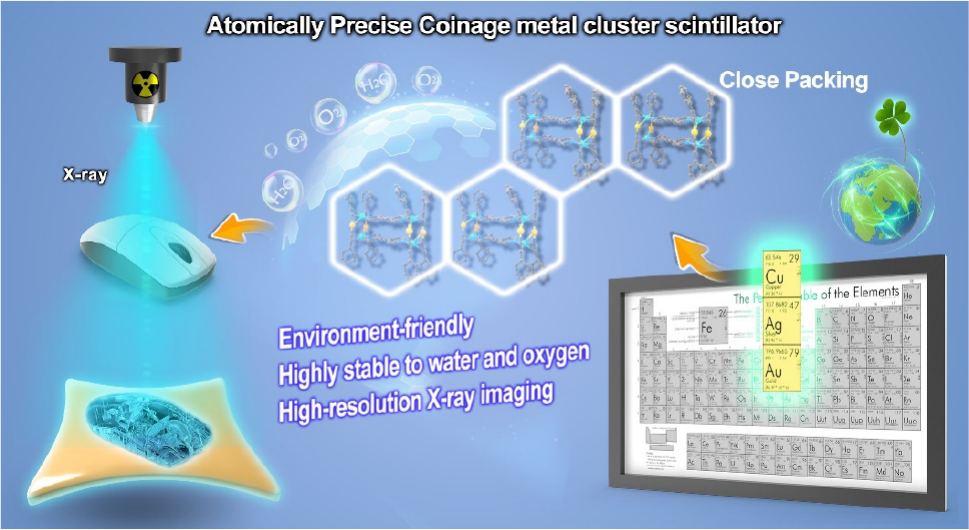

X-ray scintillators are widely used in medical imaging, industrial flaw detection, security inspection, and space exploration. However, traditional commercial scintillators are usually associated with a high use cost because of their substantial toxicity and easy deliquescence. In this work, an atomically precise Au–Cu cluster scintillator (**1**) with a thermally activated delayed fluorescence (TADF) property was facilely synthesized, which is environmentally friendly and highly stable to water and oxygen. The TADF property of **1** endows it with an ultrahigh exciton utilization rate. Combined with the effective absorption of X-ray caused by the heavy-atom effect and a limited nonradiative transition caused by close packing in the crystal state, **1** exhibits an excellent radioluminescence property. Moreover, **1** has good processability for fabricating a large, flexible thin-film device (10 cm × 10 cm) for high-resolution X-ray imaging, which can reach 40 μm (12.5 LP mm^–1^). The properties mentioned earlier make the coinage metal cluster promising for use as a substitute for traditional commercial scintillators.

## Abstract 2

Coinage metal clusters are a promising class of X-ray scintillators to substitute for traditional commercial ones in X-ray imaging due to their comprehensive advantage.

## Introduction

X-ray scintillators are a class of radiation detection materials that can convert high-energy X-ray into low-energy ultraviolet and visible light and have been widely used in medical imaging, industrial flaw detection, security inspection, and space exploration.^1−4^ The current commercial scintillators are mainly traditional inorganic semiconductors such as NaI:Tl, CsI:Tl, Bi_4_Ge_3_O_12_, PbWO_4_, and YAlO_3_:Ce, which have high radioluminescence intensities. However, inherently highly toxic metals such as Tl and Pb make traditional inorganic semiconductor scintillators a source of environmental pollution. In addition, the preparation process of these scintillators usually requires harsh conditions such as high temperature and high pressure. Moreover, the poor water-oxygen stability of these traditional inorganic semiconductor scintillators makes them difficult to store and use. Due to these challenges, traditional inorganic semiconductor scintillators have a high use cost.^5−7^ Commercial organic scintillators solved this problem to a certain extent. However, the weak X-ray absorption and radioluminescence intensity of these commercial organic scintillators due to their limited effective atomic number impede their applications very seriously. Therefore, developing new scintillators with good comprehensive advantages has become an urgent and arduous challenge.^8−10^

In recent years, organic–inorganic hybrid scintillators, especially metal–organic framework (MOF)-based scintillators, have gone in a new direction due to their highly efficient radioluminescence, good water-oxygen stability, tunable structure, and mild synthesis conditions. However, the polluting heavy metal X-ray-absorbing elements in the MOF-based scintillators are still a considerable threat to the environment. Additionally, due to some other limitations of these MOF-based scintillators, such as low synthesis efficiency and poor processability, there has been no example of their commercial application until now.^11−14^ Metal clusters are promising organic–inorganic hybrid scintillators because of their excellent photophysical properties. Moreover, due to the inherent heavy atomic core, metal clusters have a good X-ray absorption potential. Recently, Cu–I-cluster-based phosphorescence scintillators have drawn the attention of researchers, and Cu–I clusters with various radioluminescence properties have been reported successively.^15−18^ However, the phosphorescence character of these clusters causes the singlet excitons not to be used for radioluminescence effectively, limiting their exciton utilization rate. To solve this problem, introducing the thermally activated delayed fluorescence (TADF) property into the metal cluster scintillators has become a promising approach. The exciton utilization rate of scintillators with TADF properties can reach 100% theoretically.^19−22^

In this work, a Au–Cu cluster-based scintillator (**1**) with the TADF property was constructed, which could be synthesized in a one-step reaction from inexpensive starting materials at room temperature with a yield of 52%. Compared with the existing commercial scintillators, **1** not only has comparable radioluminescence intensity, an X-ray detection limit, and antiradiation stability but also has higher water-oxygen stability. The lower toxicity of coinage metals compared to that of other metals ensures **1** to be an environmentally friendly scintillator. Investigation the mechanism showed that the unique TADF process of **1** improves the utilization rate of excitons, significantly increasing the radioluminescence intensity. Meanwhile, the heavy atom effect of Au and Cu endows **1** with high X-ray absorption, and the close packing in the crystal state effectively limits the molecular thermal vibrations of **1**, blocking the nonradiative transition and further enhancing the efficiency of radioluminescence. **1** was successfully used to fabricate a flexible scintillator device with a polymer matrix, which achieved high-resolution X-ray imaging of the internal structures of various real objects. This work shows that the coinage metal cluster is expected to become a new generation of high-performance environmentally friendly scintillators to replace existing ones.

## Results and Discussion

### Synthesis and Characterization of the Scintillator

**Figure 1:**
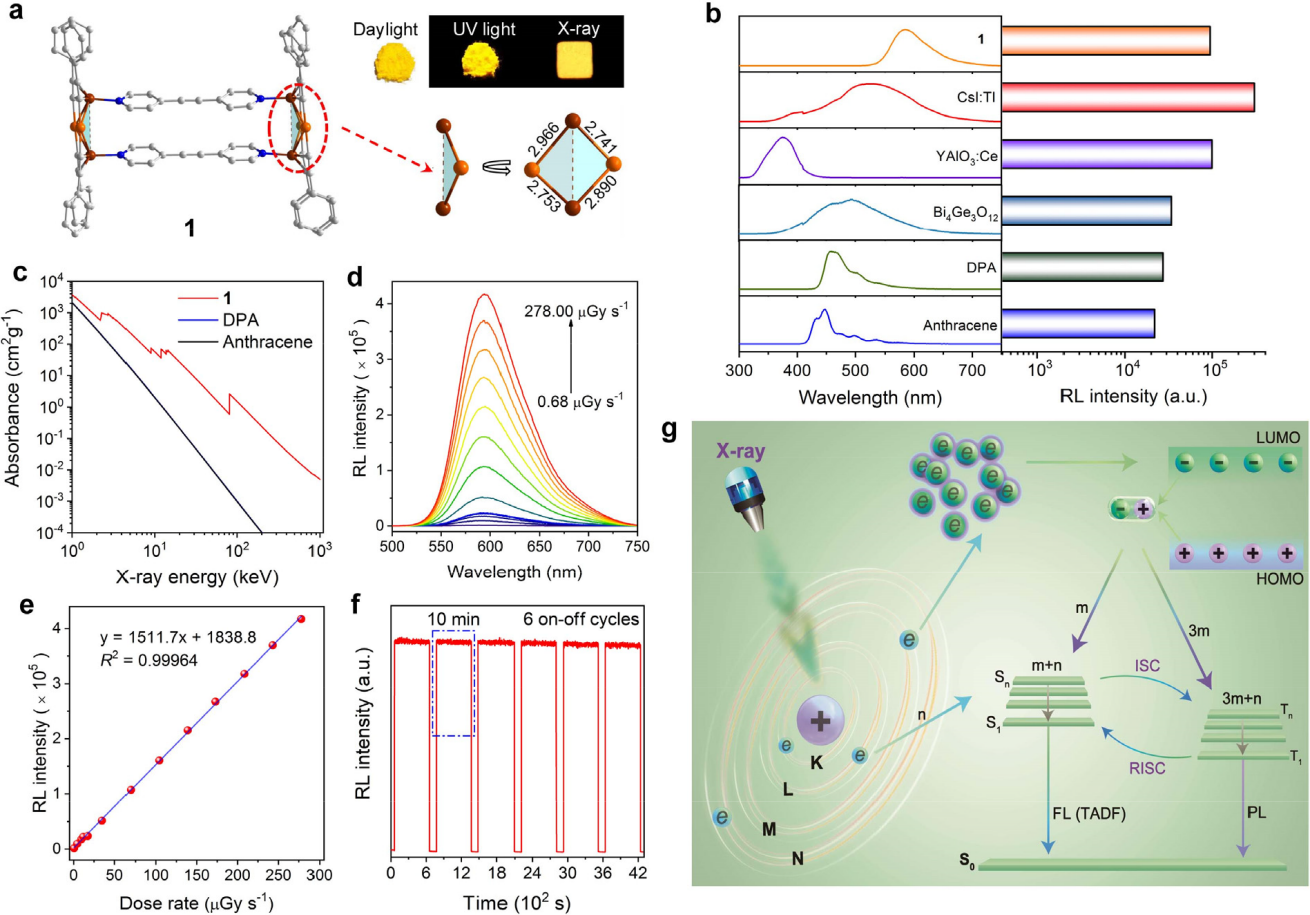
(a) Crystal structure and photographs of **1** (color codes for atoms: orange, Au; brown, Cu; blue, N; and gray, C). (b) Comparison of the radioluminescence spectra of **1** and commercial scintillators. (c) Variation of X-ray absorption with energy. (d) Dosage-dependent radioluminescence spectra of **1** in the range of 0.688–278 μGy s^–1^. (e) The dose rate dependence of the radioluminescence intensity of **1** in the range of 0.688–278 μGy s^–1^. (f) Fatigue resistance of **1** under X-ray excitation (dosage: 278 μGy s^–1^). (g) Schematic of the radioluminescence mechanism of **1** under X-ray excitation.

**1** was synthesized in situ by solvent volatilization at room temperature using 1,2-di(pyridin-4-yl)ethyne and phenylacetylene as ligands (Scheme S1). The synthetic method and structure characterizations of **1** are detailed in the Supporting Information (section 1.3). SEM images and the elemental mapping of **1** suggest that there were Au atoms and Cu atoms (Figure S1). Single-crystal X-ray diffraction analysis demonstrates that the inner core of **1** consists of space parallelograms, and the periphery is protected by the co-coordination of four phenylacetylene ligands and two 1,2-di(pyridin-4-yl)ethyne ligands (Figure 1a), resulting in a splint-shaped structure. The range of the Au–Cu bond length is 2.703–2.985 Å, which is smaller than the sum of the van der Waals radii (1.40 + 1.66 Å = 3.06 Å), indicating a strong Au–Cu affinity.^23^ Four alkynyl groups use the μ_2_-η^1^, η^2^ coordination mode. All alkynyl groups are σ-coordinated to Au atoms and π-coordinated to Cu atoms.

**1** exhibits excellent luminescence and radioluminescence properties (Figure 1a and Figures S2 and S3). The radioluminescence behavior of **1** was compared with that of commercial inorganic scintillators of CsI:Tl, YAlO_3_:Ce, and Bi_4_Ge_3_O_12_ and organic scintillators of anthracene and 9,10-diphenylanthracene (DPA) under the same conditions (Figure 1b). The radioluminescence intensity of **1** is significantly higher than that of Bi_4_Ge_3_O_12_, comparable to that of YAlO_3_:Ce, reaching 31.7% of CsI:Tl. **1** emits light in the visible region, and the full width at half-maximum values of **1** are significantly smaller than those of CsI:Tl and Bi_4_Ge_3_O_12_, which is conducive to improving the resolution of X-ray imaging. The light yield of **1** was calculated to be 26 000 photons MeV^–1^ by using the integral area ratio of the emission peak with a Bi_4_Ge_3_O_12_ crystal as reference (Figure S4).^15^ The light yields of CsI:Tl, YAlO_3_:Ce, and Bi_4_Ge_3_O_12_ are 66 000, 21 000, and 10 000 photons MeV^–1^, respectively.^24^ These results suggest that the radioluminescence properties of **1** can match the commercial inorganic scintillators. In addition, the radioluminescence intensity of **1** is significantly higher than that of commercial organic scintillators. Compared with those of anthracene and DPA, the radioluminescence intensity of **1** can reach 434.1 and 348.3%, respectively, because the Au–Cu cluster has heavier atoms with larger atomic numbers, which is better at absorbing X-rays (discussed in more detail later). As shown in Figure 1c, the X-ray absorption of **1** is higher than that of anthracene and DPA in the entire energy region (0–1000 keV).

The minimum detection limit of the X-ray dose is closely related to the radiant intensity of scintillators, which is an important indicator of the practical applications of scintillators. Thus, the relationship between the radioluminescence behavior of **1** and the X-ray dose rate was measured. As shown in Figure 1d,e, the radioluminescence intensity of **1** shows an excellent linear relationship with the X-ray dose. Moreover, the lowest detection limit for X-ray doses is 31.7 nGy s^–1^, much lower than the minimum radiation dose required for medical testing (5.5 μGy s^–1^).^25^ As can be seen in Figure 1f, **1** was exposed to a high X-ray dose rate of 278 μGy s^–1^ for six cycles of repeated excitation where each cycle duration was 10 min. The radioluminescence intensity of **1** remained at 98% of the initial intensity after 1 h of intermittent X-ray excitation, suggesting excellent antiradiation stability and fatigue resistance. As shown in Figure S5, the thermogravimetric (TG) curve and derivative thermogravimetric (DTG) curve indicate that **1** has good thermal stability within 190 °C. The PXRD data for **1** exposed to air for 1 year and immersed in water for 10 days were almost the same as for the initial crystals, demonstrating its excellent water-oxygen stability (Figure S6).

### Investigation of the Radioluminescence Mechanism

As a typical molecular-based scintillator, the radioluminescence mechanism of **1** is proposed and shown in Figure 1g.^26^ The scintillation process begins when the molecule is excited with X-rays. The entire process can be divided into four stages. In the first stage, high-energy X-ray particles are absorbed by atoms through the photoelectric effect and Compton scattering to produce high-energy electrons, while some electrons are excited to the singlet states.^27,22^ In a very short time interval, the high-energy electrons produce a large number of secondary electrons and holes through inelastic electron scattering and the Auger effect, which starts the second stage of radioluminescence. These secondary electrons and holes reside in the lowest unoccupied molecular orbital (LUMO) and the highest occupied molecular orbital (HOMO) of the molecules through thermal relaxation and then interact to produce excitons.^28,29^ In the third stage, according to the Pauli exclusion principle, the excitons are recombined in a ratio of 1:3 to form new singlet excitons and triplet excitons.^30,31^ Finally, the radiative transition processes of singlet and triplet excitons produce fluorescence and phosphorescence, respectively. As a result, high radioluminescence is usually observed with high photoluminescence. Based on this model, a series of experiments intensely studied the radioluminescence process of **1**.

The influence of heavy atoms was first investigated. A silver cluster of **2** with the highest reported photoluminescence quantum yield of 95% was used for comparison (Figure S7).^32^ As shown in Figure S8a, although **2** has intense radioluminescence, its intensity is lower than that of **1**. It may be attributed to the heavier atoms of Au compared to those of Ag, which endow **1** with better X-ray absorption.^33^ Moreover, as shown in Figure S8b, the X-ray absorption of **2** is generally inferior to that of **1** in the whole energy region. These results indicate that the interference of different metal atoms in coinage metal clusters dramatically affects their radioluminescence behaviors.

**Figure 2:**
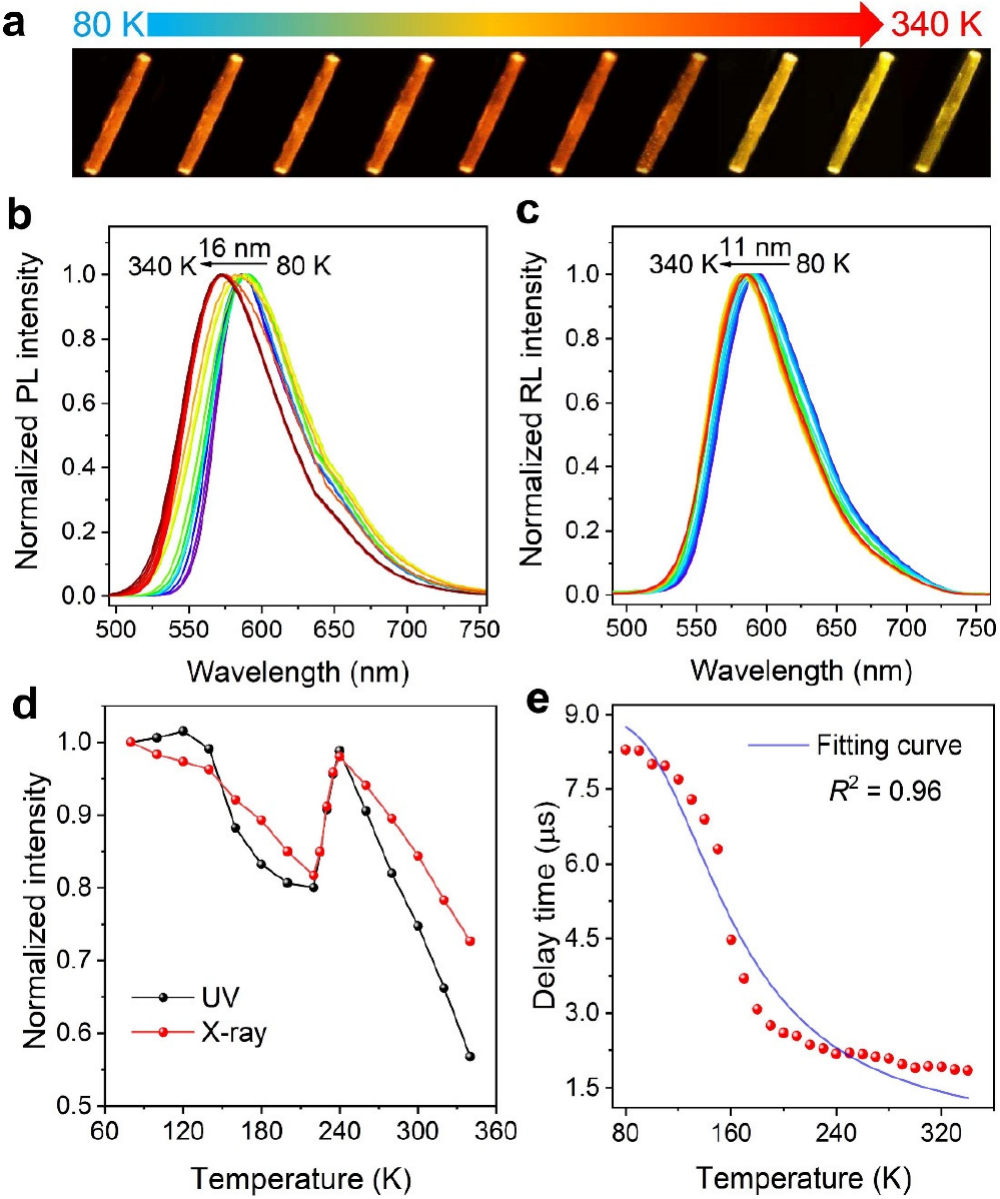
(a) Photographic image of the temperature-variable luminescence of crystal of **1** under UV light excitation. (b, c) Temperature-variable normalized emission spectra of **1** under UV light and X-ray excitation. (d) Luminescence intensity of **1** under UV light and X-ray excitation as a function of temperature. For ease of comparison, the luminescence intensities are normalized according to the data at 80 K. (e) Temperature-variable luminescence lifetimes of **1**. (The solid line is the fitting curve of the modified Boltzmann equation.)

The radiative transition process of **1** was further investigated. The temperature-variable emission spectra under UV light and X-ray excitation in the temperature range of 80–340 K were recorded to explore the mechanism of the high exciton utilization rate of **1**. As shown in Figure 2a–c, with increasing temperature, the emission wavelength of **1** was continuously blue-shifted, indicating a possible TADF process. Such a delayed process is caused by effective reverse intersystem crossing (RISC) from the lowest triple-excited state to the lowest single-excited state activated by thermal energy.^34,35^ Along with increasing temperature, the proportion of TADF (short wavelength) increased, and the proportion of phosphorescence (long wavelength) decreased. As a result, a blue shift of the emission was observed.^36,37^ Moreover, a significant emission enhancement at around 220–240 K was observed (Figure 2d and Figure S9), which is consistent with general TADF molecules.^38−40^

Next, the temperature-dependent luminescence lifetime decay curve of **1** from low to high temperatures was computed. As shown in Figure 2e, at low temperature, **1** exhibits relatively long-lasting luminescence, mainly caused by the radiative transition from the excited triplet state to the ground state. With continuous heating, the thermally activated RISC process is turned on, and the luminescence lifetime of **1** gradually decreases along with the occurrence of the TADF process, and the lifetime variation can be described by the Boltzmann equation modified through excited-state dynamics.^41,42^

The photophysical parameters of **1** were investigated in detail. The photoluminescence rate (*k*_PL_) was calculated with eq 1
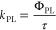
1where Φ_PL_ and τ are the photoluminescence quantum efficiency and lifetime at room temperature, respectively. For **1**, Φ_PL_***=*** 31% and τ = 1.9 μs. Thus, *k*_PL_ was calculated to be 1.6 × 10^5^ s^–1^. The phosphorescence radiative transition rate (*k*_T_1__), instantaneous fluorescence radiative transition rate (*k*_S_1__), and energy gap between the first singlet (S_1_) and triplet (T_1_) excited states (Δ*E*_ST_) were determined by fitting the temperature-dependent lifetime decay curve (Figure 2e) through the modified Boltzmann equation (eq 2)
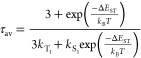
2where τ_av_, *k*_B_, and *T* are the experimental average lifetime, Boltzmann constant, and temperature, respectively.^43^ As a result, *k*_T_1__ was calculated to be 1.1 × 10^5^ s^–1^ and *k*_S_1__ was calculated to be 1.3 × 10^7^ s^–1^. *k*_PL_ is very close to *k*_T_1__, demonstrating that the radiative transition of **1** is composed of phosphorescence and TADF. In addition, Δ*E*_ST_ is calculated to be 0.05 eV, which is beneficial to the TADF process of **1** (discussed in more detail later). According to the reports, the TADF process will enhance the exciton utilization in the excited state, which should be the origin of the high photoluminescence and radioluminescence efficiency of **1**.^44,21^

**Figure 3:**
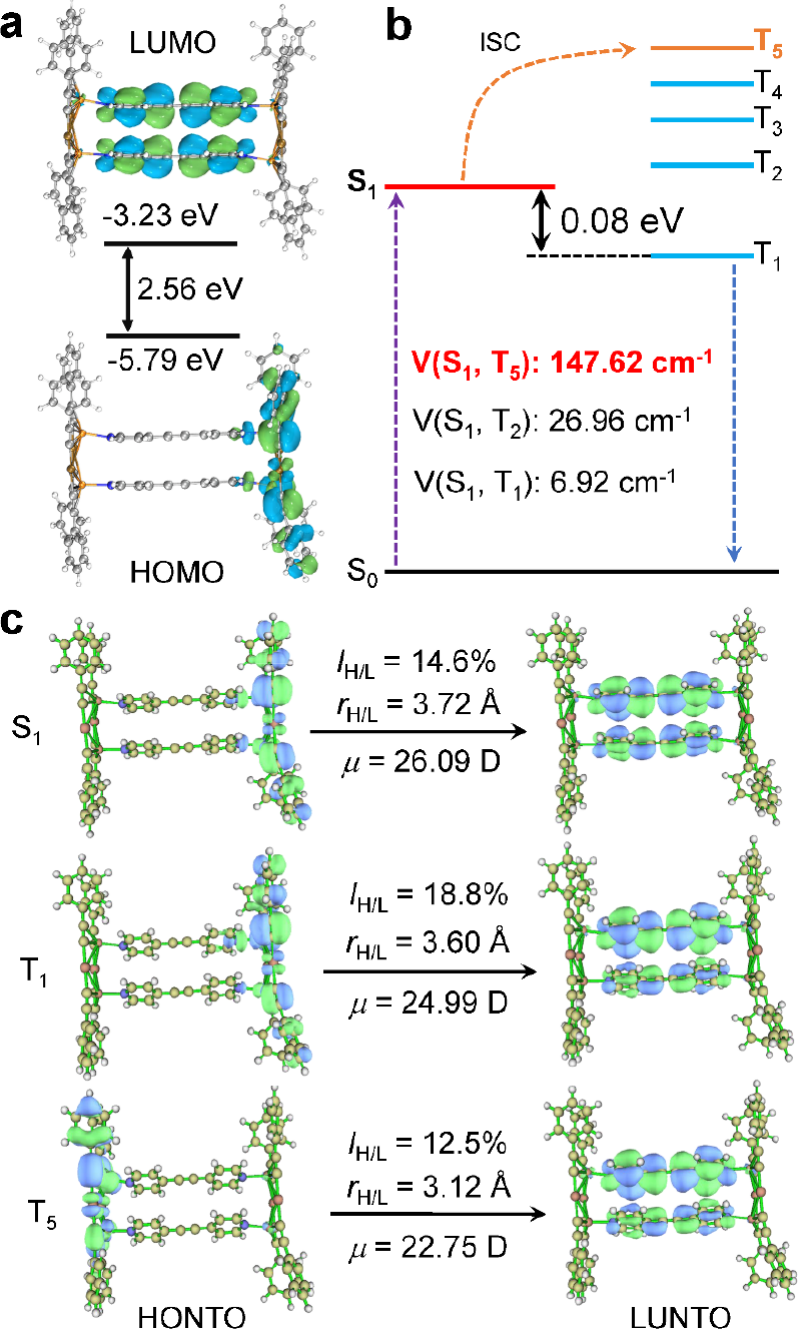
(a) Frontier molecular orbital distributions of **1** and relative energy levels of HOMO and LUMO. (b) Calculated energy diagram and SOC constants of **1**. (c) NTO analysis based on the S_0_ structures for **1**. The orbital similarity (***I***_***H***/***L***_) between HONTO and LUNTO, the charge transfer distance (***r***_***H***/***L***_), and the dipole moment variation (μ) are presented.

The ground and low-lying singlet and triplet excited states were calculated by density functional theory (DFT) and time-dependent density functional theory (TDDFT) to better understand the TADF process of **1**. As shown in Figure 3a, the HOMO and LUMO electron densities are mainly localized on the metal center and ligands, respectively. Besides, it is well known that the TADF materials rely on the high RISC components of triplet to singlet excited states. To speed up RISC, the smaller Δ*E*_ST_ between relevant singlet and triplet excited states, more significant spin–orbit coupling (SOC), and specific thermal energy would be necessary, among which the first is especially important in RISC.^45−47^ As shown in Figure 3b, the calculated Δ*E*_ST_ was 0.08 eV, which fitted well with the experimental data (0.05 eV, as mentioned earlier). The small energy gap generates a low energy barrier (smaller than 0.37 eV) between the singlet and triplet energy surfaces and enhances the electron transfer rate.^48^ Considering Δ*E*_ST_ to be proportional to the exchange integral between the singlet and triplet excited states, the orbital similarity (*I*_*H*/*L*_) of the natural transition orbitals (NTOs) was further characterized (Figure 3c). It is clear to see that **1** presents a low overlap between the highest occupied natural transition orbital (HONTO) and the lowest unoccupied natural transition orbital (LUNTO), thus generating the large dipole moment change and showing the charge transfer (CT) character.^49^ As mentioned above, the efficient TADF heavily relies on a large SOC matrix. Thus, the SOC matrices between low-lying singlet and triplet excited states were further calculated (Figure 3b). Benefiting from the heavy-atom effect, introducing Au and Cu atoms improves the SOC, which is as high as 147.62 cm^–1^. Such a large coupling is enough to drive the electron transfer between singlet and triplet potential energy surfaces.^50,51^ Similarly, the higher triplet states with large SOC matrices were also given because these higher triplet states provide the route of ISC occurring as multiple decoupling channels (Table S2).^52^ The above results indicate that the small Δ*E*_ST_ and large SOC open the availability of high exciton utilization by the TADF process.

A simplified Jablonsky-level model was constructed to further understand the difference between the luminescence process of **1** under UV light and X-ray excitation (Figure S13). The analysis of the luminescence kinetic equations shows that X-ray excitation is more favorable for the TADF process and UV light excitation is more favorable for the phosphorescence. (For the specific derivation process, see section 4 of Supporting Information.) Normally, phosphorescence is more affected by temperature than is TADF.^32^ As a result, the influence of temperature on the photoluminescence wavelength shift was larger than that of radioluminescence: 16 and 11 nm (from 80 to 340 K), respectively (Figure 2b,c).

Furthermore, the luminescence spectra of **1** and the commercial organic scintillator anthracene were compared. As shown in Figure S10, the photoluminescence intensity of **1** is weaker than that of anthracene under the same UV light excitation, while the radioluminescence intensity of **1** is much stronger than that of anthracene under the same X-ray excitation. This can be attributed to the TADF process and the heavy-atom effect of **1**. On the one hand, the exciton utilization rate of **1** is relatively stable under UV light or X-ray excitation because of the TADF process, which can effectively use both singlet and triplet excitons for radiative transition. In contrast, anthracene can use singlet excitons effectively under UV light excitation but only use 25% of singlet excitons generated by ionization excitations under X-ray excitation. The remaining 75% of triplet excitons generated by X-ray excitation are consumed as kinetic and heat energy. On the other hand, the inherent heavy atoms of Au and Cu give **1** stronger X-ray absorption compared to that of anthracene, leading to a higher radioluminescence intensity.

Moreover, the crystal structure shows that the molecules of **1** are tightly packed. There are large numbers of intramolecular and intermolecular C–H···π interactions in the stacking structure (Figure S14). The close packing prevents the direct contact of O_2_ molecules with cluster molecules, avoiding triplet state quench caused by O_2_.^53^ Meanwhile, these interactions can effectively limit the molecular thermal vibrations of **1**, blocking the nonradiative transition, enhancing the efficiency of photoluminescence and radioluminescence.^54,55^

### X-ray Imaging

**Figure 4:**
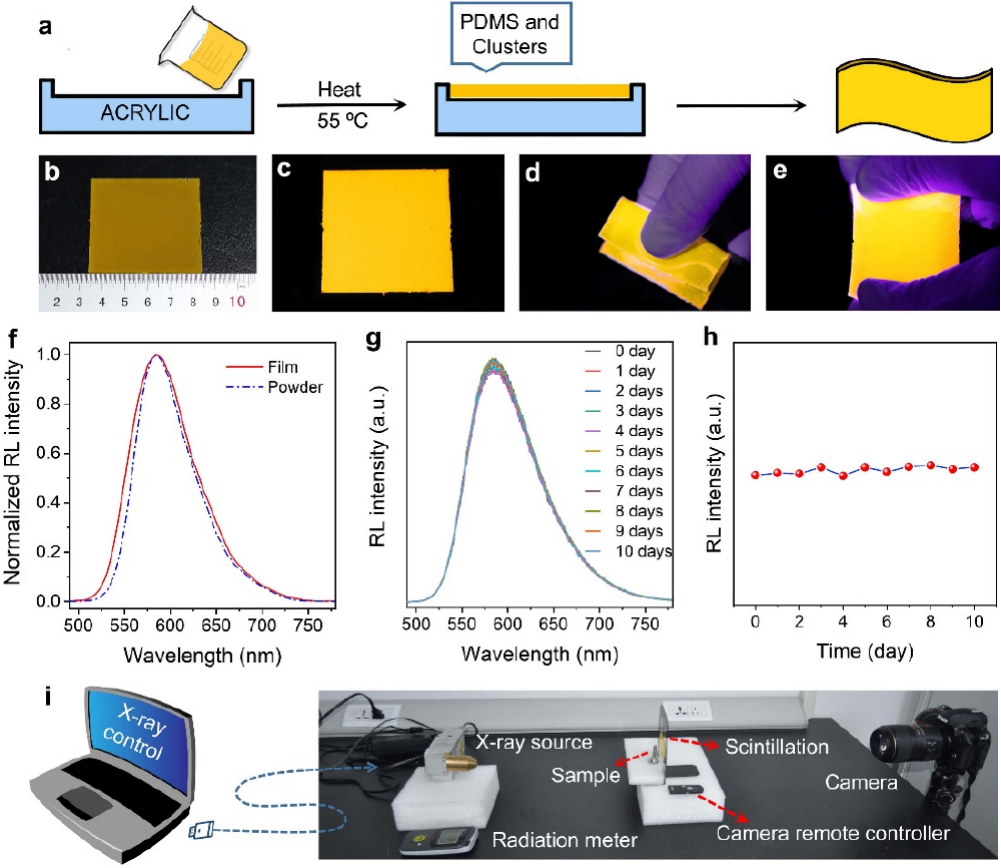
(a) Schematic diagram of the fabrication process of a flexible scintillator film. (b–e) Photographs of the scintillator film: (b) dimensions, (c) luminescence, (d) bending, and (e) stretching. (f) Normalized radioluminescence spectra of **1** powder and its PDMS film. (g) Photoluminescence spectra of a PDMS film of **1** in air for 10 days. (h) Emission intensity of a PDMS film of **1** in air for 10 days. (i) Schematic diagram of an X-ray imaging device.

Flexible devices have attracted more and more attention due to their excellent ductility, compatibility, and application value in portable wearable devices.^56^ Considering the high radioluminescence intensity and antiradiation stability, **1** was used as a background substrate to prepare a flexible scintillator film with SYLGARD 184 silicone elastomer (PDMS) (Figure 4a). As shown in Figure 4b–e, the prepared scintillator film has good flexibility. It is easy to bend and stretch and can emit uniform high-intensity light under UV light excitation. Meanwhile, the radioluminescence spectrum of the flexible film under X-ray excitation is consistent with that of the powder of **1** (Figure 4f), suggesting that **1** is still intact in the film. The stability characterization of the PDMS film was carried out and is shown in Figure 4g,h. It can be found that the radioluminescence intensity of PDMS film was almost unchanged after being kept in air for 10 days, suggesting an excellent stability of the film. Furthermore, an actual X-ray imaging application device was built (Figure 4i). The test sample was placed between the X-ray source and the coinage metal scintillator film. The image was captured directly by using a commercial digital camera.

**Figure 5:**
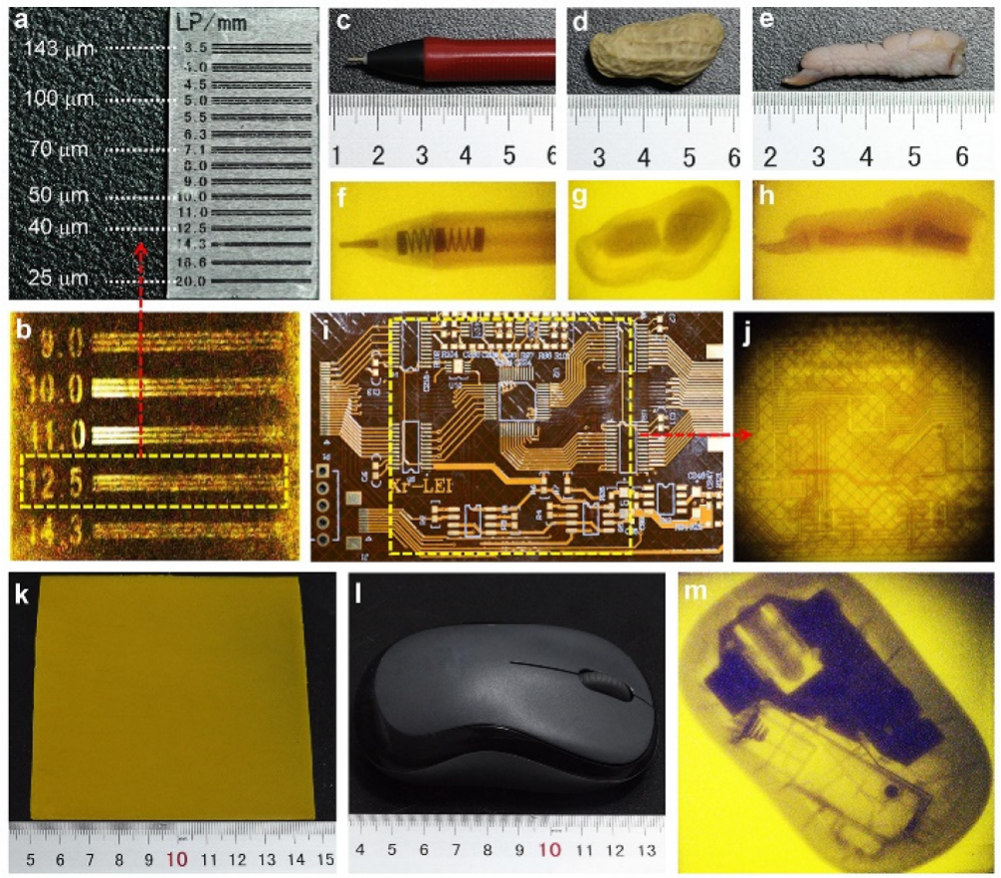
(a) X-ray high-resolution linear mask and corresponding resolution of the line pair. (b) Imaging resolution of the PDMS film of **1**. (c–j) Imaging photographs of a ballpoint pen, peanuts, chicken feet, and circuit boards. (k) Photograph of a large-scale scintillator flexible device. (l, m) Imaging pictures of the large-scale scintillator flexible device on a practical electronic device (mouse).

The X-ray imaging resolution of the scintillator film was measured using a linear mask with line widths ranging from 143 to 25 μm (Figure 5a). As shown in Figure 5b, the optimal resolution of the scintillator film of **1** can reach 40 μm (12.5 LP mm^–1^). The prepared scintillator film was used for X-ray imaging of a ballpoint pen, peanut, a chicken foot, and circuit board based on the above experiments. As shown in Figure 5c–j, under X-ray excitation the internal structure of objects can be seen on the scintillator films. In order to further demonstrate the practical application value of a coinage metal cluster scintillator in the field of flexible displays, a larger scintillator film (10 cm × 10 cm) based on **1** was prepared (Figure 5k). As shown in Figure 5l,m, the built-in electronics of the mouse can be clearly distinguished by the scintillator film under X-ray excitation. These results demonstrate that the coinage metal cluster, a new type of environmentally friendly scintillator, has good application prospects in X-ray imaging.

## Conclusions

In this work, an atomically precise Au–Cu cluster scintillator with the TADF property was constructed, which not only has a low X-ray detection limit and good antiradiation stability but also has higher water-oxygen stability compared with that of traditional commercial scintillators. In addition, the Au–Cu cluster was utilized to fabricate a flexible scintillator device with a polymer matrix, enabling high-resolution imaging of the internal structures of various real objects. More importantly, the radioluminescence mechanism of the Au–Cu cluster was researched in depth, which suggested that the intense radioluminescence originated from the ultrahigh exciton utilization rate caused by the TADF process, effective absorption of X-rays caused by the heavy-atom effect, and a limited nonradiative transition caused by close packing in the crystal state. Therefore, this work has important guiding significance for developing a new generation of high-performance, environmentally friendly X-ray scintillators.

## Supporting Information Available

Experimental details and characterizations including Figures S1–S14 and Tables S1–S4 (PDF)
Relevant crystallographic data (CIF)
checkCIF/PLATON report (PDF)

## Supplementary Material

oc3c00563_si_001.pdf

oc3c00563_si_002.cif

## Accession Codes

CCDC 2208177 contain the supplementary crystallographic data for this paper. These data can be obtained free of charge via www.ccdc.cam.ac.uk/data_request/cif, or by emailing data_request@ccdc.cam.ac.uk, or by contacting The Cambridge Crystallographic Data Centre, 12 Union Road, Cambridge CB2 1EZ, UK; fax: + 44 1223 336033.
