# Supplementary material for: Thermally Activated Delayed Fluorescence Coinage Metal Cluster Scintillator
Source: ACS Cent Sci. 2023 Jun 24;9(7):1419–26. doi: 10.1021/acscentsci.3c00563 (PMC10375876; doi:10.1021/acscentsci.3c00563)
Supplement: Supplementary file 1 — oc3c00563_si_001.pdf [file oc3c00563_si_001.pdf]

Supporting Information for

Thermally Activated Delayed Fluorescence Coinage Metal Cluster Scintillator

Qiu-Chen Peng,[a] Yu-Bing Si,[a] Zhao-Yang Wang,[a] Shu-Heng Dai,[b] Qiu-Shui Chen,[b] Kai Li,*[a] Shuang-Quan Zang*[a]

[a] Henan Key Laboratory of Crystalline Molecular Functional Materials, Henan International Joint Laboratory of Tumor Theranostical Cluster Materials, Green Catalysis Center and College of Chemistry, Zhengzhou University, Zhengzhou 450001, China.

[b] MOE Key Laboratory for Analytical Science of Food Safety and Biology, State Key Laboratory of Photocatalysis on Energy and Environment, College of Chemistry, Fuzhou University, Fuzhou 350100, China.

* Email: zangsqzg@zzu.edu.cn; likai@zzu.edu.cn;

Table of Contents

1. Experimental..S3

1.1 Reagents..S3

1.2 Apparatus..S3

1.3 Synthesis...S4

1.4 X-Ray crystallography...S5

1.5 X-ray emission measurements..S5

1.6 X-ray dosage detection...S6

1.7 Fabrication of flexible scintillation devices...S6

1.8 Flexible X-ray luminescence imaging...S7

2. Selected spectra and data referred in the paper...S8

3. Theoretical calculation...S14

4. Luminescence kinetics of photoluminescence and radioluminescence............S16

5. Crystal structures...S19

6. References...S22

1. Experimental

1.1 Reagents

Unless otherwise stated, all raw materials of analytical grade were commercially available and do not need to be purified. 1,2-di(pyridin-4-yl)ethyne were purchased from Yanshen Technology Co., Ltd, Jinlin, China. Phenylacetylene, chloroauric acid and tetrakis(acetonitrile)copper(I) hexa-fluorophosph were purchased from Energy Chemical Co., Shanghai, China. Commercial scintillators of CsI:TI, $YAlO_3$:Ce, $Bi_4Ge_3O_{12}$, anthracene and 9, 10-diphenylanthracene was purchased from TCI Chemical Co., Shanghai, China. SYLGARD 184 silicone elastomer (containing curing agent) was purchased from Alpha Chemical Co., Zhengzhou, China. All the other materials including solvents of analytical grade, and phosphate buffer were purchased from Sinopharm Chemical Reagent Beijing Co., Beijing, China.

1.2 Apparatus

Rigaku MiniFlex diffractometer (Cu-Kα; λ = 1.54178 Å; 2θ range of 3-50°) was used to collect the PXRD data in air at room temperature. Scanning electron microscope (SEM) and elemental mapping measurements were collected via Zeiss Sigma 500. Thermogravimetry analysis (TGA) was performed by a SHIMADZU TGA-Q50 thermogravimetric analyzer under N_2 atmosphere with a heating rate of 10 °C/min. The elemental analysis data was obtained on a Thermo Flash EA 1112 Analyzer. Edinburgh FLS-980 fluorescence spectrometer with integrating sphere ($BaSO_4$) was used to collect the luminescence spectra, lifetimes, and absolute quantum yields. UV-Vis diffuse reflectance spectra were measured by an Agilent Cary 5000 UV-Vis-NIR Spectrometer using $BaSO_4$ as the reference. X-ray excited luminescent spectra were recorded using an Edinburgh FS5 fluorescence spectrophotometer (Edinburgh Instruments Ltd.) with a miniature X-ray source (Amptek Inc.). Photographs of X-ray or UV-induced luminescence and radioluminescence-based X-ray imaging were acquired with a digital camera (Nikon, D850 coupled with AF-S Micro-Nikkor 105 mm 2.8G or AF-S Micro-Nikkor 40 mm 2.8G) in an all-manual mode.

1.3 Synthesis

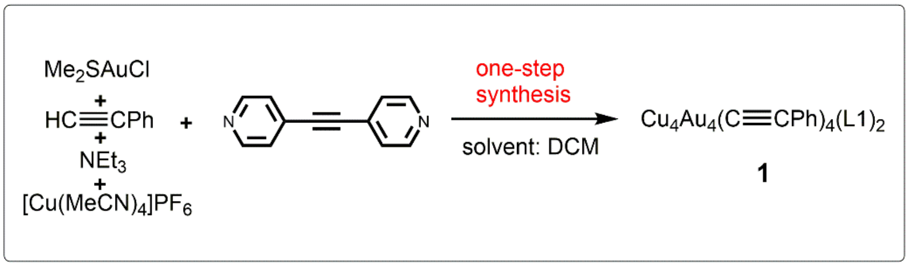

Scheme S1. Synthetic route of **1**.

Synthesis of Me_2SAuCl

$H[AuCl_4]\cdot 3H_2O$ (1.00 g, 2.5 mmol) was dissolved in EtOH (25 mL) and to this was added SMe_2 (600 μL, 8.2 mmol) in EtOH (25 mL) with rapid stirring and a white precipitate began to form. The solution was stirred at room temperature for 2 hours until no color remained. The solution was cooled to 0 °C and the product isolated by filtration, washing with cold EtOH (3 × 10 mL) and dry diethyl ether (3 × 10 mL). The product was dried in vacuo to yield a white solid (0.74 g, 95%).

Synthesis of 1

Me_2SAuCl (60 mg, 0.2 mmol) was dissolved in 10 mL of dichloromethane, then 100 mL of phenylacetylene and 100 mL of triethylamine were added, the color of the solution first changed from colorless to yellow, shake gently, and then changed back to colorless. Tetrakis(acetonitrile)copper(I)hexa-fluorophosph (75 mg, 0.2 mmol) was dissolved in 20 mL of dichloromethane and slowly added to the colorless solution, the color of the solution changed from colorless to bright orange. Then 1,2-di(pyridin-4-yl)ethyne (60 mg, 0.33 mmol) was dissolved in 10 mL of dichloromethane and slowly added to the bright orange solution, the color of the solution changed from bright orange to cyan. After 30 minutes of evaporation at room temperature, orange striped crystals were formed in the solution. The crystals were collected by filtration and washed with dichloromethane. Yield: 52% (based on Au). Elemental analysis calcd. (%) for $C_{88}H_{56}Au_4Cu_4N_4$: C 47.79; H 2.55; N 2.53; found: C 47.80; H 2.42; N 2.38.

1.4 X-ray crystallography

Single-crystal X-ray diffraction (SCXRD) measurement of **1** was performed on a Rigaku XtaLAB Pro diffractometer with Cu-Kα radiation (λ = 1.5418 Å) at 200 K. Data collection and reduction was performed using the program CrysAlisPro.[1] The structure was solved with direct method (SHELXS)[2] and refined by full-matrix least squares on F^2 using OLEX2,[3] which utilizes the SHELXL-2015 module.[4] All the atoms were refined anisotropically. Hydrogen atoms were placed in calculated positions refined using idealized geometries and assigned fixed isotropic displacement parameters. Structure refinement was handled with different strategies according to the electron density distribution. The detailed information of the crystal data and refinement results are summarized in Table S3-S4.

1.5 X-ray emission measurements

The radioluminescence spectra of **1** with different sample thicknesses have been measured (Figure S11). As shown in Figure S11b, the thickness of the sample can affect its radioluminescence intensity, but the effect is small. The radioluminescence intensity of the sample with a thickness of 0.5 mm is 87% of that with a thickness of 5 mm. Although thicker sample can absorb more X-ray, corresponding stronger self-absorption will also restrict its radioluminescence intensity. As a result, the radioluminescence intensity reached its maximum value at a thickness of 4 mm. Thus, all the scintillators are put in the same sample cell, which make them has same surface and thickness (4 mm).

All the measurements are carried out under the same miniature X-ray source (Amptek Inc.), the X-ray dosage was 278 μGy s^{-1}, all the settings such as slit, temperature, etc., are same. Based on this method, the radioluminescence intensities of the samples are compared. The data of X-ray absorbance of the materials was generated by calculation from a publicly available database (the web link is https://physics.nist.gov/PhysRefData/Xcom/html/xcom1.html, access date: 2023-6-6).

1.6 X-ray dosage detection

X-ray luminescence spectra of **1** were obtained with varied X-ray dosage from 0.688 μGy s^{-1} to 278.0 μGy s^{-1}. The radiation dose of X-ray is controlled by changing the current and voltage of miniature X-ray source. The relationship between the current and voltage of miniature X-ray source and the corresponding radiation dose is shown in Table S1. The limit of detection (LOD) was calculated by LOD = $\frac{3\sigma}{k}$, where σ is the standard deviation calculated by repeated tests of background signals for ten times, and k is the slope of linear fitting curve.

Table S1. Relationship between dose rate and voltage/current of X-ray source.

Voltage (kV)	Current (μA)	Dose rate (μGy s^{-1})
10	5	0.68844
20	5	4.5830
30	5	9.6410
40	5	11.912
50	5	17.375
50	10	34.750
50	20	69.500
50	30	104.250
50	40	139.000
50	50	173.750
50	60	208.500
50	70	243.250
50	80	278.000

1.7 Fabrication of a flexible scintillation device

SYLGARD 184 silicone elastomer and curing agent was premixed in a mass ratio of 10:1. **1** (5 *wt* %) was uniformly dispersed in the n-hexane solution, and then added to the premixed polymer matrix. The mixture was stirred vigorously to form the mixture gel. The gel mixture was placed in a plexiglas mold (5 cm × 5 cm × 1 mm or 10 cm × 10 cm × 1 mm) and heated for 5 hours at 65 °C using a high-precision glue maker. When the mixture gel was cooled to room temperature, it was peeled from the mold to form smooth and flat scintillator films.

1.8 Flexible X-ray luminescence imaging

To evaluate the imaging performance of **1**, a high-resolution X-ray imaging system was constructed. The system consists of miniature X-ray source, scintillator films, and a digital camera. The operating voltage and current of the micro X-ray light tube are 0 - 200 kV and 0 - 60 μA, respectively. During the imaging process, the sample was placed between the X-ray source and scintillator films, and the digital camera was used to collect pictures from the other side of the scintillator film. The actual picture of the device is shown in Figure 4i.

2. Selected spectra and data referred in the paper

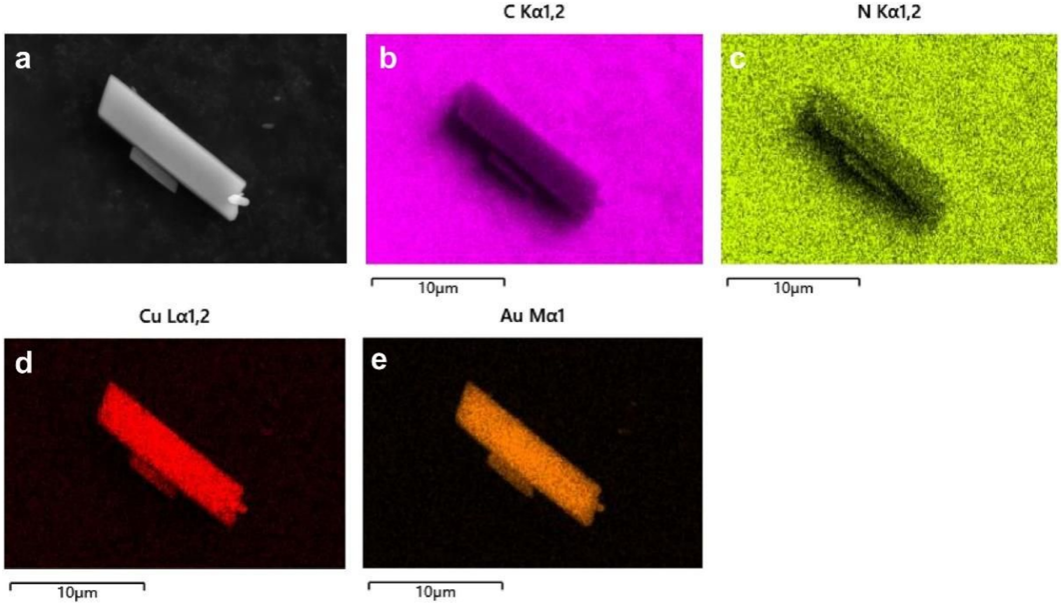

Figure S1. SEM images and elemental mapping of **1**.

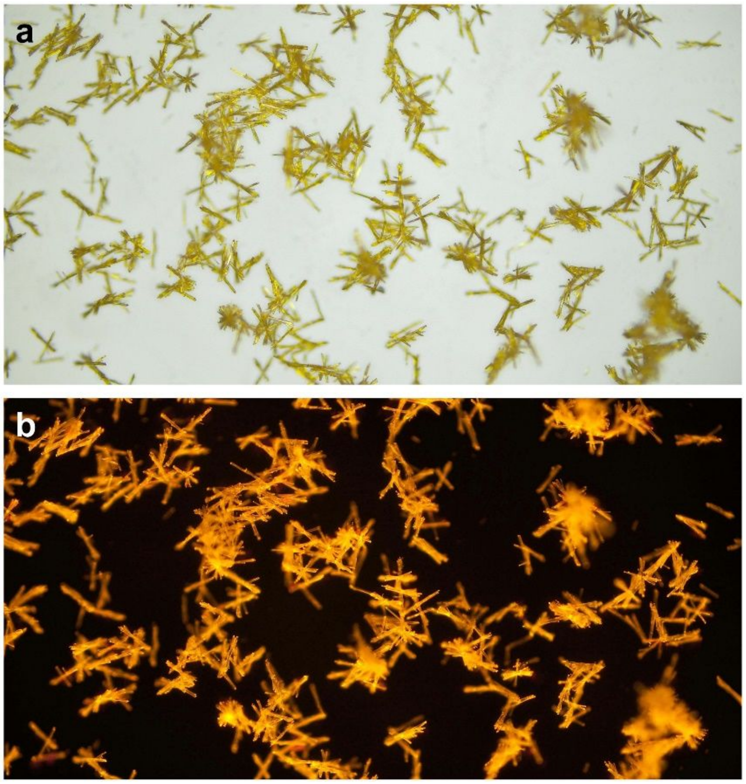

Figure S2. Photographic image of large number of crystals of **1** under (a) day light and (b) UV light.

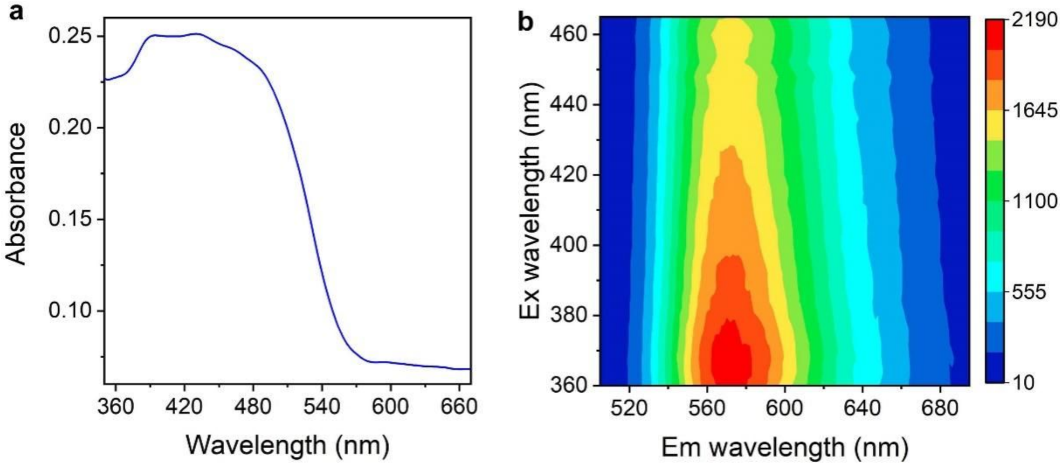

Figure S3. (a) UV-Vis diffuse reflectance spectra of **1**. (b) 3D luminescence spectra of **1**.

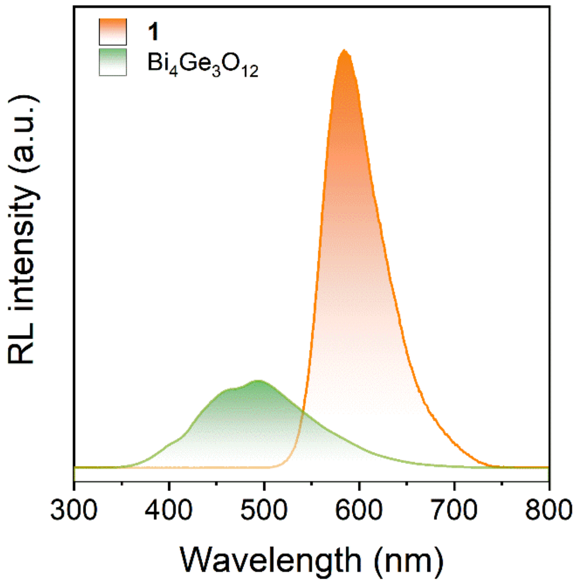

Figure S4. Radioluminescence spectra of **1** and $Bi_4Ge_3O_{12}$ single crystals under the same X-ray illumination.

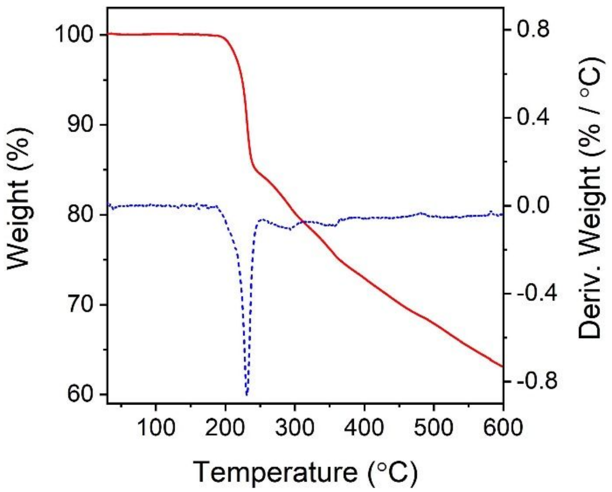

Figure S5. The TG curve and DTG curve of **1**.

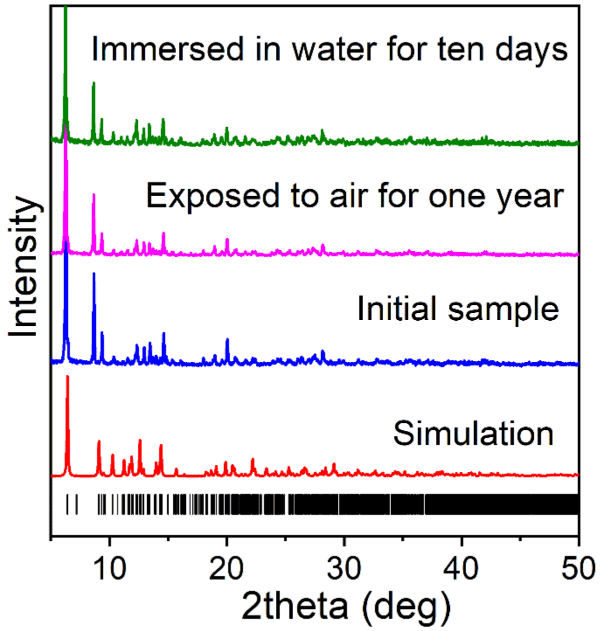

Figure S6. PXRD of **1** exposed to air for one year and soaked in water.

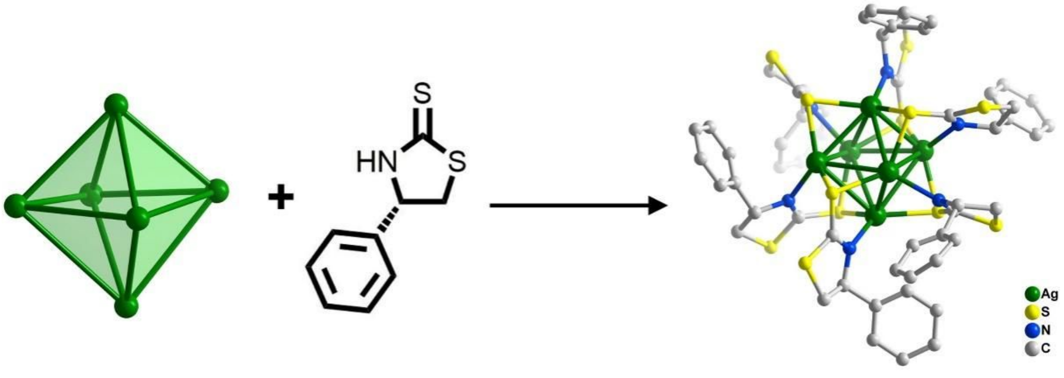

Ag_6 **2**

Figure S7. Crystal structure of **2**.

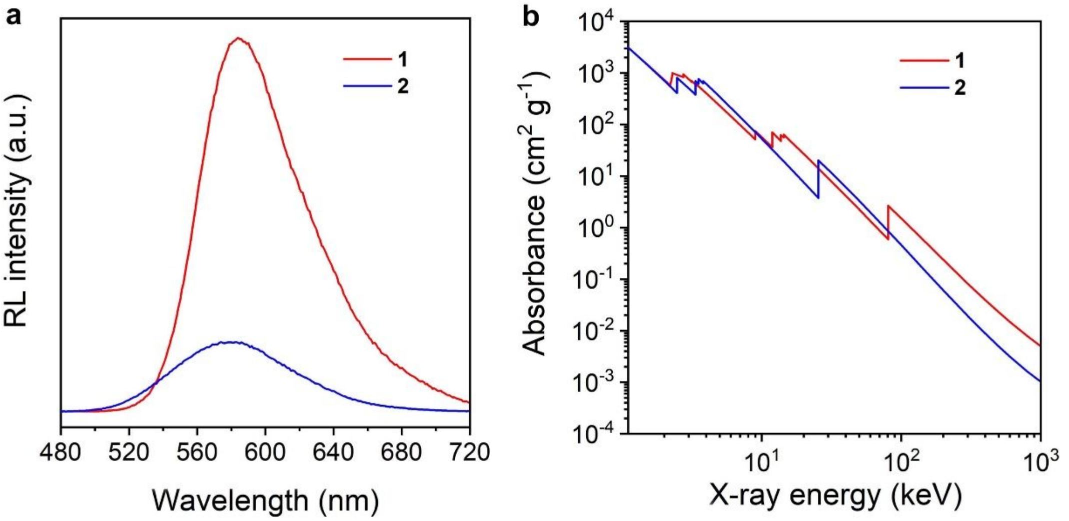

Figure S8. (a) Emission spectra of **1** and **2** under X-ray excitation. (b) The variation of X-ray absorption with energy.

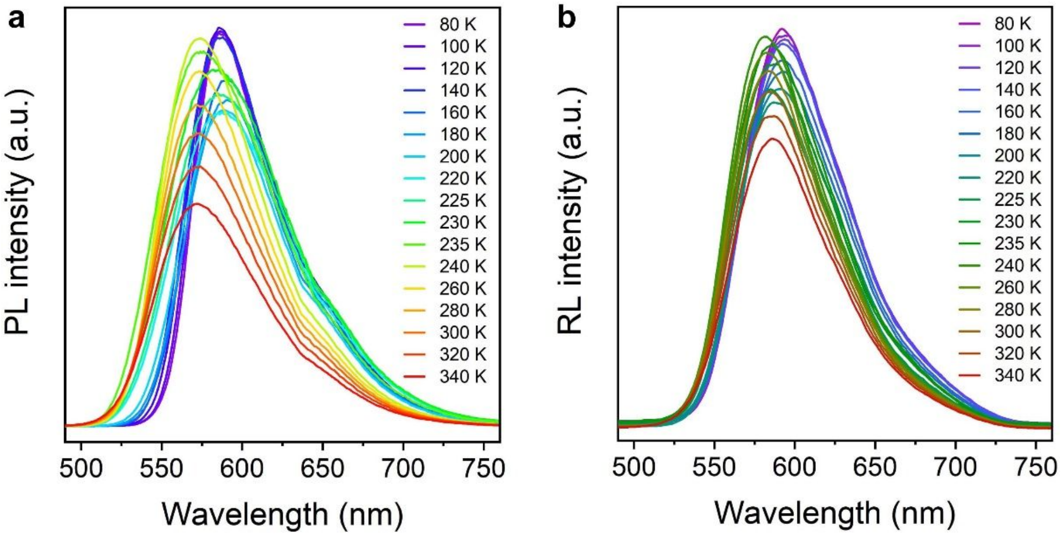

Figure S9. (a) Temperature-variable emission spectra of **1** under UV light excitation. (b) Temperature-variable emission spectra of **1** under X-ray excitation.

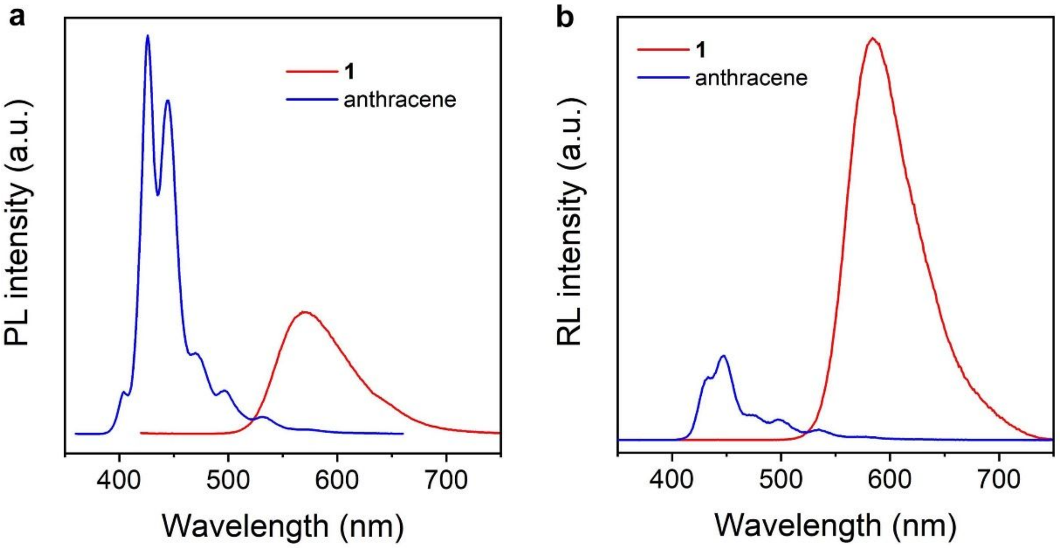

Figure S10. Emission spectra of **1** and anthracene under UV light and X-ray excitation.

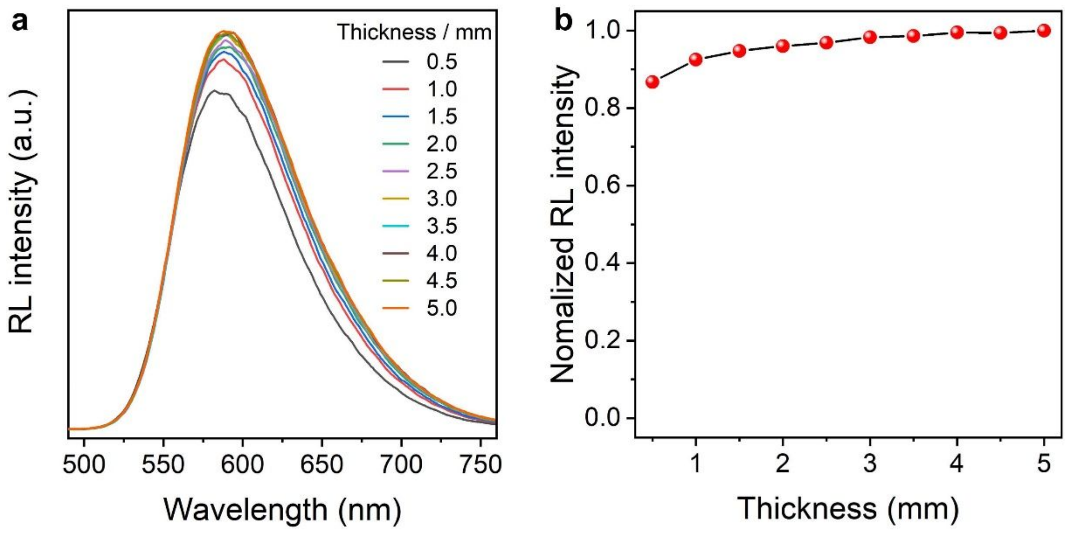

Figure S11. (a) Radioluminescence spectra of **1** with different thickness. (b) Radioluminescence intensity of **1** as a function of thickness. For ease of comparison, the radioluminescence intensities are normalized according to the data of 5 mm.

3. Theoretical calculations

The ground state and singlet and triplet excited states were calculated by density functional theory (DFT) and time-dependent density functional theory (TDDFT) approaches. The model was built from the single-crystal structure of **1**. The two-layer ONIOM scheme for quantum mechanics/molecular mechanics (QM/MM) calculations was implemented to deal with the electronic structure of crystal (Figure S12).[5,6] One organic scintillator involved in the center of the crystal structures was chosen as "QM layer" and calculated at the PBE0/def2-SVP level, while the others molecules surrounded are treated as "MM layer" and calculated by the universal force field (UFF).[7] The electronic embedding scheme is used in the QM/MM calculations to incorporate the partial charges of the MM region into the QM Hamiltonian. The geometry optimization of ground and excited states were calculated by Gaussian 16 package.[8] All the spin-orbit coupling calculations were simulated by ORCA 5.0.3 program[9] with the PBE0 functional in conjunction with the large scalar relativistically reconstructed DKH-def2-SVP basis set (SARC-DKH-TZVP for Au atoms) and the matching auxiliary basis set of SARC/J was used.[10] The 3D plot of the orbital overlap integral mapping and natural transition orbitals (NTOs) of the low-lying excited states were generated and visualized by Multiwfn and VMD programs.[11,12]

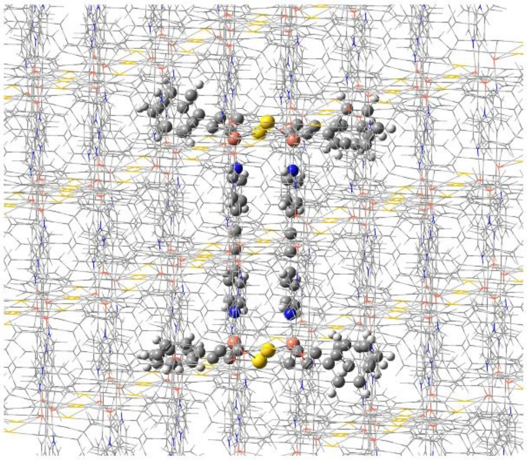

Figure S12. Hybrid QM/MM model of **1**. QM and MM molecules are represented ball/stick and line models, respectively.

Table S2. Calculated spin-orbit coupling matrix elements between singlet and triplet excited states for **1**.

S	T	MS = 0	MS = 1	MS = -1	$\langle S|\hat{H}_{SO}|T\rangle$ (cm^{-1})
1	1	(0.00, -1.72)	(4.31, 1.98)	(4.31, -1.98)	6.92
1	2	(0.00, 21.17)	(11.58, -2.27)	(11.58, 2.27)	26.96
1	3	(0.00, -21.35)	(-18.94, -2.10)	(-18.94, 2.10)	39.85
1	4	(0.00, -50.96)	(-23.98, -7.94)	(-23.98, 7.94)	62.23
1	5	(0.00, 93.65)	(80.54, 4.90)	(80.54, -4.90)	147.62
1	6	(0.00, -25.67)	(-50.14, -25.99)	(-50.14, 25.99)	83.89
1	7	(0.00, -54.74)	(-10.50, 31.51)	(-10.50, -31.51)	72.13
1	8	(0.00, 2.51)	(0.43, 0.67)	(0.43, -0.67)	2.75
1	9	(0.00, 11.04)	(9.71, -1.64)	(9.71, 1.64)	17.77
1	10	(0.00, 6.73)	(-20.28, -1.29)	(-20.28, 1.29)	29.52

4. Luminescence kinetics of photoluminescence and radioluminescence

To understand the difference between photoluminescence and radioluminescence in luminescence kinetics, we constructed a simple Jablonsky energy level model as shown in Figure S13.

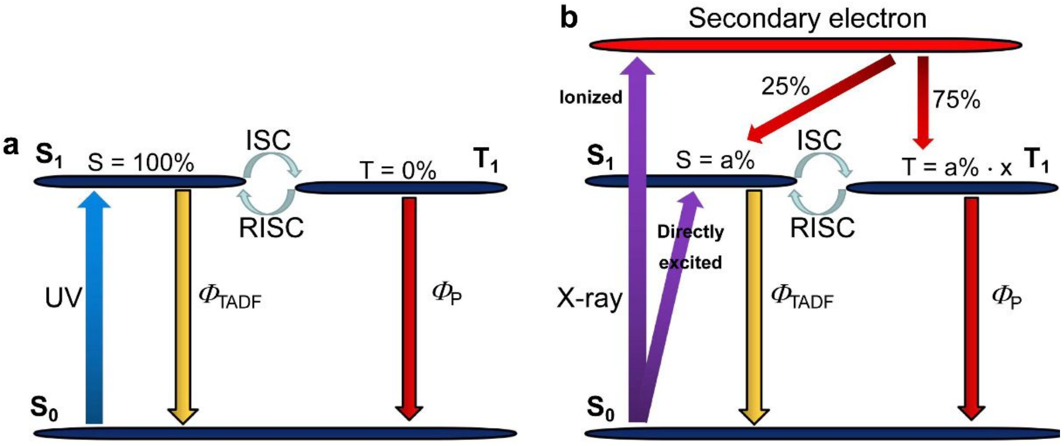

Figure S13. The simple Jablonsky energy level model of photoluminescence and radioluminescence of **1**.

Under UV excitation, for scintillators with TADF properties, the relationship between phosphorescence efficiency (Φ_{P}), intersystem transit efficiency (Φ_{ISC}), reverse intersystem transit efficiency (Φ_{RISC}), thermal activation delayed fluorescence efficiency (Φ_{TADF}) and total luminescence efficiency (Φ_{PL}) is as follows:

$$\Phi_{PL} = \Phi_{P} + \Phi_{TADF} \quad (1)$$

Considering the dynamic balance between the singlet state and the triplet state:

$$\Phi_{TADF} = \lim_{n\sim\infty} \sum_{i=1}^{n} \Phi_{P} \cdot (\Phi_{ISC}\Phi_{RISC})^{n} \quad (2)$$

Bring equation (2) into formula equation (1):

$$\Phi_{PL} = \Phi_{P} \cdot \lim_{n\sim\infty} \sum_{i=0}^{n} (\Phi_{ISC}\Phi_{RISC})^{n} = \Phi_{P} \cdot \lim_{n\sim\infty} \frac{1-(\Phi_{ISC}\Phi_{RISC})^{n}}{1-\Phi_{ISC}\Phi_{RISC}}$$

Because the number of loops *n* tends to be positive infinity, hence:

$$\Phi_{PL} = \frac{\Phi_P}{1 - \Phi_{ISC}\Phi_{RISC}} \quad (3)$$

Bring equation (3) into equation (1):

$$\Phi_{TADF} = \frac{\Phi_{ISC}\Phi_{RISC}}{1 - \Phi_{ISC}\Phi_{RISC}} \cdot \Phi_P \quad (4)$$

From equation (1) we can get:

$$\frac{\Phi_{TADF}}{\Phi_{PL}} = \frac{\Phi_{TADF}}{\Phi_{TADF} + \Phi_P} \quad (5)$$

Bring equation (4) into equation (5):

$$\frac{\Phi_{TADF}}{\Phi_{PL}} = \frac{\frac{\Phi_{ISC}\Phi_{RISC}}{1 - \Phi_{ISC}\Phi_{RISC}}\Phi_P}{\frac{\Phi_{ISC}\Phi_{RISC}}{1 - \Phi_{ISC}\Phi_{RISC}}\Phi_P + \Phi_P} = \Phi_{ISC}\Phi_{RISC} = A$$

The first cycle is special due to the direct generation of singlet excitons and triplet excitons under X-ray excitation. After the first intersystem crossing and intersystem crossing, the percentages of singlet excitons and triplet excitons change from the original S ($0 < S < 1$) and T ($0 < T < 1$) to S' ($0 < S' < 1$) and T' ($0 < T' < 1$), which satisfy the following relationship:

$$T = x \cdot S \ (x > 1) \quad (6)$$

$$T' = (\Phi_{ISC} + x) \cdot S \quad (7)$$

$$S' = (\Phi_{ISC} + x) \cdot S \cdot \Phi_{RISC} \quad (8)$$

The following infinite number of cycles of dynamic equilibrium is the same as that of UV excitation. Therefore, the phosphorescence efficiency under X-ray irradiation (Φ_P^X), the thermally activated delayed fluorescence efficiency (Φ_{TADF}^X), the total luminescence Efficiency (Φ_{PL}^X) satisfies the following relationship:

$$\Phi_{PL}^X = \Phi_P^X + \Phi_{TADF}^X \quad (9)$$

$$\Phi_P^X = T \cdot \Phi_P \quad (10)$$

$$\Phi_{TADF}^X = \lim_{n\sim\infty} \sum_{i=1}^{n} T' \cdot \Phi_P \cdot (\Phi_{ISC}\Phi_{RISC})^n = \lim_{n\sim\infty} T' \cdot \Phi_P \cdot \frac{1 - (\Phi_{ISC}\Phi_{RISC})^n}{1 - \Phi_{ISC}\Phi_{RISC}}$$

Because the number of loops *n* tends to be positive infinity, hence:

$$\Phi_{TADF}^{X} = \frac{T' \cdot \Phi_P}{1 - \Phi_{ISC}\Phi_{RISC}} \quad (11)$$

Bring equation (7) into equation (11):

$$\Phi_{TADF}^{X} = \frac{(\Phi_{ISC} + x) \cdot S \cdot \Phi_P}{1 - \Phi_{ISC}\Phi_{RISC}} \quad (12)$$

From equation (9) we can get:

$$\frac{\Phi_{TADF}^{X}}{\Phi_{PL}^{X}} = \frac{\Phi_{TADF}^{X}}{\Phi_{P}^{X} + \Phi_{TADF}^{X}} \quad (13)$$

Bring Equation (6) and Equation (12) into Equation (13):

$$\frac{\Phi_{TADF}^{X}}{\Phi_{PL}^{X}} = \frac{\frac{(\Phi_{ISC} + x) \cdot S \cdot \Phi_P}{1 - \Phi_{ISC}\Phi_{RISC}}}{\frac{(\Phi_{ISC} + x) \cdot S \cdot \Phi_P}{1 - \Phi_{ISC}\Phi_{RISC}} + x \cdot S \cdot \Phi_P} = \frac{\Phi_{ISC} + x}{\Phi_{ISC} + x + 1 - \Phi_{ISC}\Phi_{RISC}} = B$$

From the above derivation we can get:

$$B - A = \frac{\Phi_{ISC} + x}{\Phi_{ISC} + x + 1 - \Phi_{ISC}\Phi_{RISC}} - \Phi_{ISC}\Phi_{RISC} = \frac{\Phi_{ISC} + \Phi_{ISC}\Phi_{RISC} + (\Phi_{ISC}\Phi_{RISC} + x) \cdot (1 - \Phi_{ISC}\Phi_{RISC})}{\Phi_{ISC} + x + (1 - \Phi_{ISC}\Phi_{RISC})}$$

Because:

$$x > 1; 0 < \Phi_{ISC} < 1; 0 < \Phi_{RISC} < 1$$

Therefore:

$$B - A > 0$$

Which is:

$$\frac{\Phi_{TADF}^{X}}{\Phi_{PL}^{X}} > \frac{\Phi_{TADF}}{\Phi_{PL}} \quad (14)$$

Bring equation (1) and equation (9) into equation (1) we can get:

$$\frac{\Phi_{P}^{X}}{\Phi_{PL}^{X}} < \frac{\Phi_{P}}{\Phi_{PL}} \quad (15)$$

In conclusion, X-ray excitation is more favorable for the TADF process of scintillators with TADF properties.

5. Crystal structure

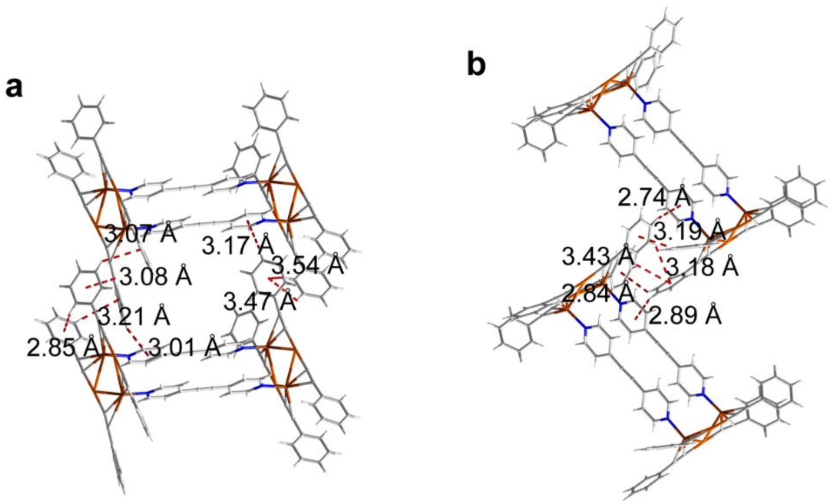

Figure S14. Crystal packing modes of **1** (red dotted line indicates C-H···π action).

Table S3. Crystal data and structure refinements of **1**.

	**1**
Empirical formula	$C_{88}H_{56}Au_4Cu_4N_4$
Formula weight	2211.39
Temperature/K	200
Crystal system	triclinic
Space group	*P*-1
a/Å	9.5278(3)
b/Å	13.7473(4)
c/Å	27.6689(9)
α/°	89.931(2)
β/°	82.873(3)
γ/°	87.336(2)
Volume/Å3	3592.19(2)
Z	2
ρcalcg/cm^3	2.004
μ/mm^{-1}	16.598
F(000)	2088.0
Crystal size/mm^3	0.15×0.11×0.1
Radiation	CuKα (λ = 1.54184 Å)
2θrange for data collection/°	6.436 to 148.724
Index ranges	-11 ≤ h ≤ 11, -17 ≤ k ≤ 17, -17 ≤ l ≤ 34
Reflections collected	35476
Independent reflections	14039 [R_{int} = 0.0638, R_{sigma} = 0.0803]
Data/restraints/parameters	14039/201/969
Goodness-of-fit on F^2	1.060
Final R indexes [I>=2σ (I)]	R_1 = 0.0526, wR_2 = 0.1281
Final R indexes [all data]	R_1 = 0.0822, wR_2 = 0.1408
CCDC	2208177

Table S4. Bond lengths of **1**

Atom-Atom	Bond length (Å)
Au1-Cu1	2.7030(15)
Au1-Cu2	2.9122(14)
Au1-C25	2.013(11)
Au1-C41	2.042(11)
Au2-Cu1	2.9851(14)
Au2-Cu2	2.7229(15)
Au2-C33	2.022(10)
Au2-C49	2.013(11)
Au3-Cu3	2.7528(15)
Au3-Cu4	2.9640(15)
Au3-C65	2.013(11)
Au3-C86	2.013(10)
Au4-Cu3	2.8900(15)
Au4-Cu4	2.7416(16)
Au4-C57	2.002(14)
Au4-C73	1.999(14)
Cu1-N1	2.063(7)
Cu1-C41	2.052(10)
Cu1-C49	2.105(9)
Cu1-C50	2.356(10)
Cu2-N3	2.033(7)
Cu2-C25	2.091(9)
Cu2-C26	2.310(10)
Cu2-C33	2.091(10)
Cu3-N2	2.030(7)
Cu3-C57	2.073(11)
Cu3-C58	2.332(11)
Cu3-C86	2.105(10)
Cu3-N4	2.056(8)
Cu4-C65	2.102(10)
Cu4-C66	2.375(10)
Cu4-C73	2.042(11)

6. References

[1] CrysAlisPro 2012, Agilent Technologies. Version 1.171.36.31.

[2] Sheldrick, G. M. A short history of *SHELX*. *Acta Cryst. A*, 2008, **64**, 112.

[3] Dolomanov, O. V., Bourhis, L. J., Gildea, R. J., Howard, J. A. K. & Puschmann, H. *OLEX2*: acomplete structure solution, refinement and analysis program. *J. Appl. Cryst.*, 2009, **42**, 339.

[4] Sheldrick, G. M. Crystal structure refinement with *SHELXL*. *Acta Cryst. C*, 2015, **71**, 3.

[5] Dapprich, S., Komáromi, I., Byun, K. S., Morokuma, K. & Frisch, M. J. A new ONIOM implementation in Gaussian 98. 1. The calculation of energies, gradients and vibrational frequencies and electric field derivatives. *J. Mol. Struct. (Theochem)*., 1999, **462**, 1-21.

[6] Vreven, T., Morokuma, K., Farkas, Ö., Schlegel, H. B. & Frisch, M. J. Geometry optimization with QM/MM, ONIOM and other combined methods. I. Microiterations and constraints. *J. Comp. Chem*., 2003, **24**, 760-769.

[7] Rappé, A. K., Casewit, C. J., Colwell, K. S., III, W. A.G. & Skiff, W. M. UFF, a full periodic-table force-field for molecular mechanics and molecular-dynamics simulations. *J. Am. Chem. Soc*., 1992, **114**, 10024-10035.

[8] Gaussian 16., Revision A.03., Frisch, M. J., Trucks, G. W., Schlegel, H. B., Scuseria, G. E., M. A. Robb, M. A., Cheeseman, J. R., Scalmani, G., Barone, V., Petersson, G. A. & H. Nakatsuji, H. Gaussian, Inc., Wallingford CT, 2016.

[9] Neese, F., Wennmohs, F., Becker, U. & Riplinger, C. The ORCA quantum chemistry program package. *J Chem Phys*., 2020, **14**, 224108.

[10] Rolfes, D. J., Neese, F. J. & Pantazis, D. A. All-electron scalar relativistic basis sets for the elements Rb-Xe. *J. Comput. Chem*., 2020, **41**, 1842-1849.

[11] Lu, T., Chen, F. Multiwfn: a multifunctional wavefunction analyzer. *J. Comput. Chem*., 2012, **33**, 580-592.

[12] Humphrey, W., Dalke, A. & Schulten, K. VMD-visual molecular dynamics. *J. Molec. Graphics*., 1996, **14**, 33-38.
