# Supplementary material for: Thermally Activated Delayed Fluorescence Coinage Metal Cluster Scintillator
Source: ACS Cent Sci. 2023 Jun 24;9(7):1419–26. doi: 10.1021/acscentsci.3c00563 (PMC10375876; doi:10.1021/acscentsci.3c00563)

checkCIF/PLATON report

Structure factors have been supplied for datablock(s) hy

THIS REPORT IS FOR GUIDANCE ONLY. IF USED AS PART OF A REVIEW PROCEDURE FOR PUBLICATION, IT SHOULD NOT REPLACE THE EXPERTISE OF AN EXPERIENCED CRYSTALLOGRAPHIC REFEREE.

No syntax errors found. CIF dictionary Interpreting this report

Datablock: hy

```
Bond precision:   C-C = 0.0157 A          Wavelength=1.54184

Cell:           a=9.5278(3)        b=13.7473(4)      c=27.6689(9)
                alpha=89.931(2)    beta=82.873(3)    gamma=87.336(2)
Temperature:    200 K
```

	Calculated	Reported
Volume	3592.2(2)	3592.19(19)
Space group	P -1	P -1
Hall group	-P 1	-P 1
Moiety formula	C88 H56 Au4 Cu4 N4	C88 H56 Au4 Cu4 N4
Sum formula	C88 H56 Au4 Cu4 N4	C88 H56 Au4 Cu4 N4
Mr	2211.45	2211.39
Dx,g cm-3	2.045	2.044
Z	2	2
Mu (mm-1)	16.598	16.598
F000	2088.0	2088.0
F000’	2040.75	
h,k,lmax	11,17,34	11,17,34
Nref	14659	14039
Tmin,Tmax	0.168,0.190	0.626,1.000
Tmin’	0.055	

```
Correction method= # Reported T Limits: Tmin=0.626 Tmax=1.000
AbsCorr = MULTI-SCAN

Data completeness= 0.958            Theta(max)= 74.362

R(reflections)= 0.0526( 9667)       wR2(reflections)= 0.1408( 14039)

S = 1.060                   Npar= 969
```

The following ALERTS were generated. Each ALERT has the format
test-name_ALERT_alert-type_alert-level.
Click on the hyperlinks for more details of the test.

Alert level C

```
PLAT234_ALERT_4_C Large Hirshfeld Difference C63      --C64      .       0.16 Ang.
PLAT234_ALERT_4_C Large Hirshfeld Difference C69      --C70      .       0.17 Ang.
PLAT342_ALERT_3_C Low Bond Precision on  C-C Bonds ..............    0.01574 Ang.
PLAT370_ALERT_2_C Short  C(sp2)-C(sp1) Bond  C25      - C26      .       1.18 Ang.
PLAT370_ALERT_2_C Short  C(sp2)-C(sp1) Bond  C49      - C50      .       1.19 Ang.
PLAT370_ALERT_2_C Short  C(sp2)-C(sp1) Bond  C57      - C58      .       1.20 Ang.
PLAT370_ALERT_2_C Short  C(sp2)-C(sp1) Bond  C65      - C66      .       1.18 Ang.
PLAT370_ALERT_2_C Short  C(sp2)-C(sp1) Bond  C73      - C74      .       1.20 Ang.
PLAT906_ALERT_3_C Large K Value in the Analysis of Variance ......     11.452 Check
PLAT906_ALERT_3_C Large K Value in the Analysis of Variance ......      2.448 Check
PLAT911_ALERT_3_C Missing FCF Refl Between Thmin & STh/L=    0.600        102 Report
PLAT972_ALERT_2_C Check Calcd Resid. Dens.  0.82Ang From Au3          -2.23 eA-3
PLAT972_ALERT_2_C Check Calcd Resid. Dens.  0.74Ang From Au2          -2.02 eA-3
PLAT972_ALERT_2_C Check Calcd Resid. Dens.  0.94Ang From Au2          -2.02 eA-3
PLAT972_ALERT_2_C Check Calcd Resid. Dens.  0.83Ang From Au1          -2.00 eA-3
PLAT972_ALERT_2_C Check Calcd Resid. Dens.  0.70Ang From Au4          -1.72 eA-3
PLAT972_ALERT_2_C Check Calcd Resid. Dens.  0.91Ang From Au4          -1.67 eA-3
PLAT972_ALERT_2_C Check Calcd Resid. Dens.  0.82Ang From Au2          -1.66 eA-3
PLAT972_ALERT_2_C Check Calcd Resid. Dens.  0.89Ang From Au1          -1.62 eA-3
PLAT972_ALERT_2_C Check Calcd Resid. Dens.  0.84Ang From Au3          -1.62 eA-3
PLAT972_ALERT_2_C Check Calcd Resid. Dens.  0.92Ang From Au3          -1.60 eA-3
PLAT977_ALERT_2_C Check Negative Difference Density on H21       .      -0.31 eA-3
PLAT977_ALERT_2_C Check Negative Difference Density on H96       .      -0.36 eA-3
```

Alert level G

```
PLAT002_ALERT_2_G Number of Distance or Angle Restraints on AtSite         17 Note
PLAT003_ALERT_2_G Number of Uiso or Uij Restrained non-H Atoms ...         22 Report
PLAT172_ALERT_4_G The CIF-Embedded .res File Contains DFIX Records          2 Report
PLAT174_ALERT_4_G The CIF-Embedded .res File Contains FLAT Records          1 Report
PLAT177_ALERT_4_G The CIF-Embedded .res File Contains DELU Records          2 Report
PLAT186_ALERT_4_G The CIF-Embedded .res File Contains ISOR Records          2 Report
PLAT301_ALERT_3_G Main Residue  Disorder ..............(Resd  1  )        10% Note
PLAT343_ALERT_2_G Unusual sp?      Angle Range in Main Residue for        C33 Check
PLAT343_ALERT_2_G Unusual sp?      Angle Range in Main Residue for        C41 Check
PLAT343_ALERT_2_G Unusual sp?      Angle Range in Main Residue for        C86 Check
PLAT371_ALERT_2_G Long   C(sp2)-C(sp1) Bond  C3       - C6       .       1.41 Ang.
PLAT371_ALERT_2_G Long   C(sp2)-C(sp1) Bond  C7       - C8       .       1.41 Ang.
PLAT371_ALERT_2_G Long   C(sp2)-C(sp1) Bond  C15      - C18      .       1.43 Ang.
PLAT371_ALERT_2_G Long   C(sp2)-C(sp1) Bond  C19      - C20      .       1.44 Ang.
PLAT371_ALERT_2_G Long   C(sp2)-C(sp1) Bond  C26      - C27      .       1.47 Ang.
PLAT371_ALERT_2_G Long   C(sp2)-C(sp1) Bond  C34      - C35      .       1.45 Ang.
PLAT371_ALERT_2_G Long   C(sp2)-C(sp1) Bond  C42      - C43      .       1.46 Ang.
PLAT371_ALERT_2_G Long   C(sp2)-C(sp1) Bond  C50      - C51      .       1.44 Ang.
PLAT371_ALERT_2_G Long   C(sp2)-C(sp1) Bond  C58      - C59      .       1.45 Ang.
PLAT371_ALERT_2_G Long   C(sp2)-C(sp1) Bond  C66      - C67      .       1.47 Ang.
PLAT371_ALERT_2_G Long   C(sp2)-C(sp1) Bond  C74      - C75      .       1.44 Ang.
PLAT371_ALERT_2_G Long   C(sp2)-C(sp1) Bond  C87      - C88      .       1.45 Ang.
PLAT860_ALERT_3_G Number of Least-Squares Restraints ............        201 Note
PLAT910_ALERT_3_G Missing # of FCF Reflection(s) Below Theta(Min).          1 Note
```

```
PLAT912_ALERT_4_G Missing # of FCF Reflections Above STh/L=  0.600        517 Note
PLAT933_ALERT_2_G Number of HKL-OMIT Records in Embedded .res File          4 Note
PLAT941_ALERT_3_G Average HKL Measurement Multiplicity ..........         2.5 Low
PLAT978_ALERT_2_G Number C-C Bonds with Positive Residual Density.          0 Info
```

```
   0 ALERT level A = Most likely a serious problem - resolve or explain
   0 ALERT level B = A potentially serious problem, consider carefully
  23 ALERT level C = Check. Ensure it is not caused by an omission or oversight
  28 ALERT level G = General information/check it is not something unexpected

   0 ALERT type 1 CIF construction/syntax error, inconsistent or missing data
  36 ALERT type 2 Indicator that the structure model may be wrong or deficient
   8 ALERT type 3 Indicator that the structure quality may be low
   7 ALERT type 4 Improvement, methodology, query or suggestion
   0 ALERT type 5 Informative message, check
```

It is advisable to attempt to resolve as many as possible of the alerts in all categories. Often the minor alerts point to easily fixed oversights, errors and omissions in your CIF or refinement strategy, so attention to these fine details can be worthwhile. In order to resolve some of the more serious problems it may be necessary to carry out additional measurements or structure refinements. However, the purpose of your study may justify the reported deviations and the more serious of these should normally be commented upon in the discussion or experimental section of a paper or in the "special_details" fields of the CIF. checkCIF was carefully designed to identify outliers and unusual parameters, but every test has its limitations and alerts that are not important in a particular case may appear. Conversely, the absence of alerts does not guarantee there are no aspects of the results needing attention. It is up to the individual to critically assess their own results and, if necessary, seek expert advice.

Publication of your CIF in IUCr journals

A basic structural check has been run on your CIF. These basic checks will be run on all CIFs submitted for publication in IUCr journals (*Acta Crystallographica*, *Journal of Applied Crystallography*, *Journal of Synchrotron Radiation*); however, if you intend to submit to *Acta Crystallographica Section C* or *E* or *IUCrData*, you should make sure that full publication checks are run on the final version of your CIF prior to submission.

Publication of your CIF in other journals

Please refer to the *Notes for Authors* of the relevant journal for any special instructions relating to CIF submission.

PLATON version of 19/02/2022; check.def file version of 19/02/2022

Datablock hy - ellipsoid plot

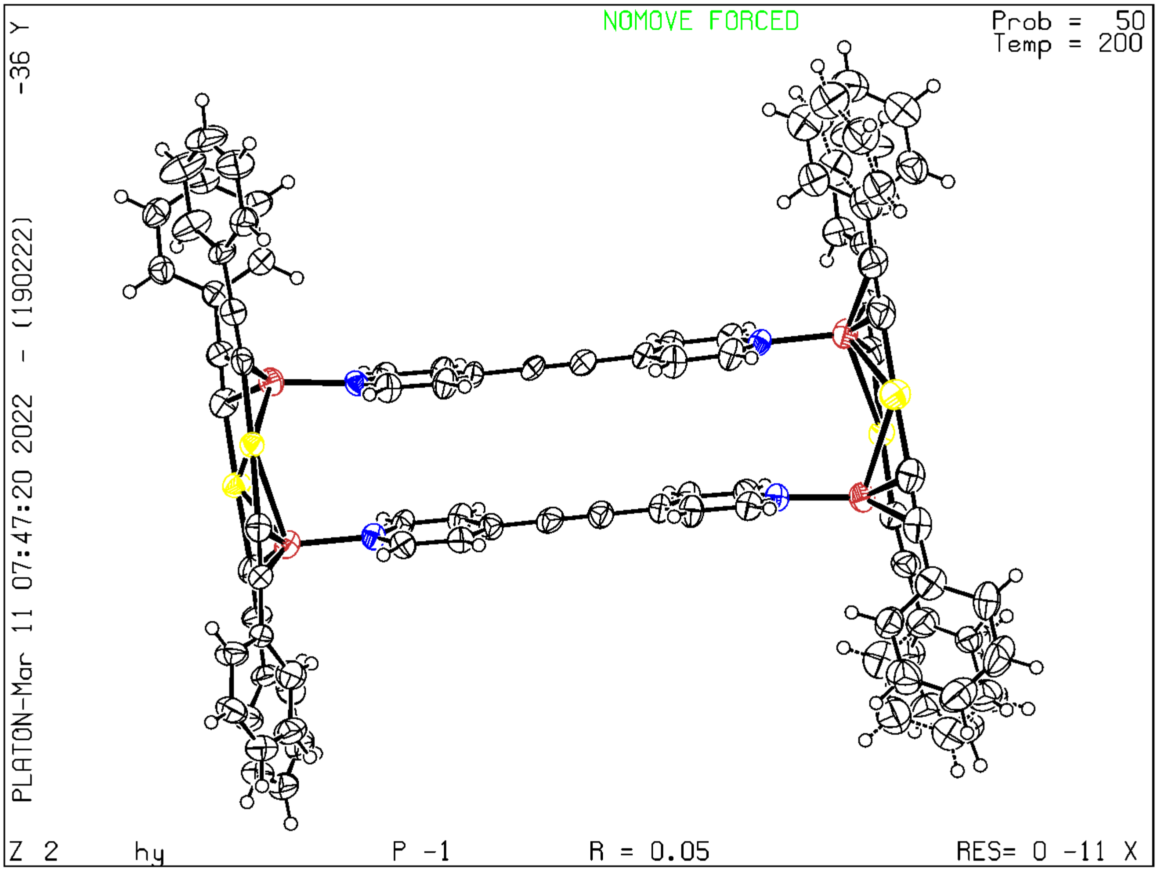

Supplement: Supplementary file 3 — oc3c00563_si_003.pdf [file oc3c00563_si_003.pdf]
